# Determination of the electrostatic potential distribution in Pt/Fe:SrTiO_3_/Nb:SrTiO_3_ thin-film structures by electron holography

**DOI:** 10.1038/srep06975

**Published:** 2014-11-10

**Authors:** Astrid Marchewka, David Cooper, Christian Lenser, Stephan Menzel, Hongchu Du, Regina Dittmann, Rafal E. Dunin-Borkowski, Rainer Waser

**Affiliations:** 1 Institut für Werkstoffe der Elektrotechnik II, RWTH Aachen University, Sommerfeldstr. 24, 52074 Aachen, Germany; 2 CEA LETI, 17 rue des Martyrs, 38054 Grenoble Cedex 9, France; 3 Peter Grünberg Institut, Forschungszentrum Jülich GmbH, 52425 Jülich, Germany; 4 Ernst-Ruska-Centrum für Mikroskopie und Spektroskopie mit Elektronen, Forschungszentrum Jülich GmbH, 52425 Jülich, Germany

## Abstract

We determined the electrostatic potential distribution in pristine Pt/Fe:SrTiO_3_/Nb:SrTiO_3_ structures by electron holography experiments, revealing the existence of a depletion layer extending into the Nb-doped bottom electrode. Simulations of potential profiles in metal-insulator-metal structures were conducted assuming different types and distributions of dopants. It is found that the presence of acceptor-type dopant concentrations at the Fe:SrTiO_3_/Nb:SrTiO_3_ interface with a donor-doped insulating layer provides a good match to the measured profile. Such acceptor-type interface concentrations may be associated with Sr vacancies on the Nb:SrTiO_3_ side of the bottom interface.

Metal-insulator-metal (MIM) structures based on transition metal oxide thin films have attracted tremendous attention as resistance switching memory cells for next-generation nonvolatile memory applications^1^,^2^,^3^. These cells can be reversibly switched between a high resistive and a low resistive state with appropriate electrical stimuli. In asymmetric structures that contain one Schottky-like and one ohmic metal-insulator contact, the Schottky-like interface is believed to play an essential role in the switching process as well as in the initial electroforming process. Electroformation of a pristine sample is typically required to establish the switchable state and turns the initially insulating structure into a more conductive state. The underlying mechanism is proposed to rely on the growth of conductive oxygen vacancy filaments, altering the barrier at the Schottky-like interface^4^,^5^,^6^. The switching process is thought to involve a reversible change in barrier height and width by redistribution of oxygen vacancies near the Schottky-like interface^7^,^8^,^9^ or by charging and discharging of interface traps^10^,^11^. In the model system consisting of epitaxial SrTiO_3_ thin films grown on conducting Nb-doped SrTiO_3_ single crystals that also serve as the bottom electrode^12^, it is generally assumed that the bottom interface can be regarded as an ohmic contact, with switching taking place at the top interface^13^,^14^. However, evidence of the exact potential distribution and of the shape of the barrier in the structures of interest is missing.

Phase contrast techniques in the transmission electron microscope (TEM) such as electron holography^15^ or Fresnel contrast analysis^16^ allow for capturing local changes in the electrostatic potential and have been previously applied to analyse the electrostatic potential at different interfaces, e. g. in undoped and Nb-doped SrTiO_3_ bicrystals^16^,^17^, grain boundaries in SrTiO_3_^18^, or Si *p*-*n* junctions^19^,^20^.

In this study, we measure the electrostatic potential profile across a pristine Pt/Fe-doped SrTiO_3_/Nb-doped SrTiO_3_ layer stack using off-axis electron holography and use numerical simulations to interpret the results. A good match to the experimental potential distribution is provided by introducing acceptor-type dopants at the bottom electrode interface with a donor-doped oxide layer in the simulations. For the application of Pt/Fe:SrTiO_3_/Nb:SrTiO_3_ structures as resistance switching elements, we conclude that the local field distribution may affect the site of oxygen vacancy movement and needs to be taken into account when analysing resistive switching phenomena.

## Results

**Figure 1:**
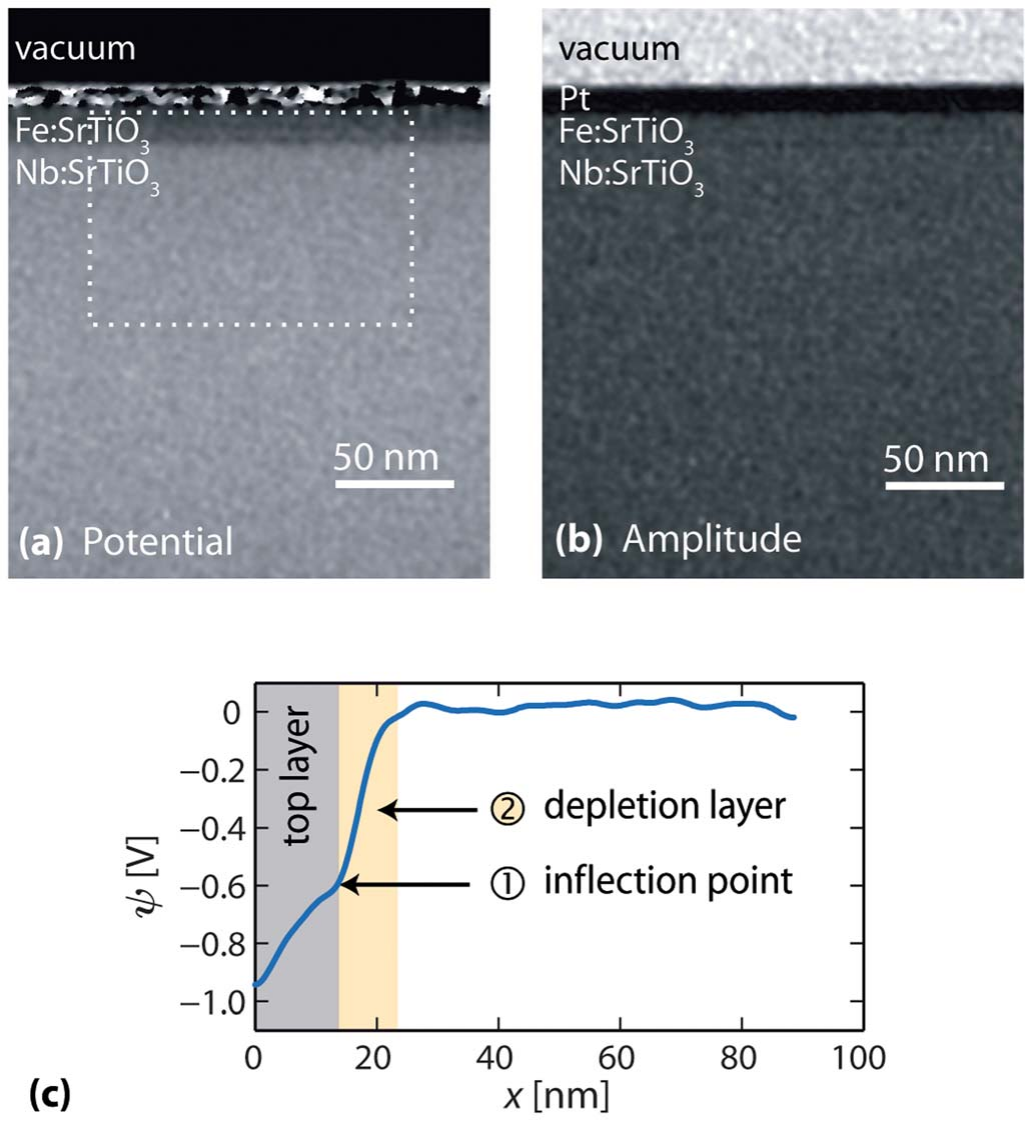
(a) Reconstructed phase image of a Pt/Fe:SrTiO_3_/Nb:SrTiO_3_ sample; (b) Reconstructed amplitude image showing the homogeneity of the sample and absence of strong diffraction contrast; (c) Extracted electrostatic potential averaged across the region indicated in (a).

Off-axis electron holography was used to determine the potential distribution across the Pt/Fe:SrTiO_3_/Nb:SrTiO_3_ layer stack. The technique involves using an electron biprism to interfere an electron wave that has passed through the region of interest with a reference wave that has passed only through vacuum. The resulting interference pattern (or electron hologram) is analysed to obtain a real-space representation of the phase of the electrons that have passed through the specimen. The phase, in turn, is proportional to the electrostatic potential within and around the specimen projected in the electron beam direction^21^ and the recorded holograms can be converted into maps of the change in potential across the layers using the relation^15^

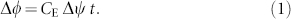
Here, *φ* is the phase, *C*_E_ is a constant that depends on the microscope accelerating voltage (0.0073 rads V^−1^ nm^−1^ at 200 kV), *ψ* is the potential, and *t* is the specimen thickness. Figure 1(a) shows an electrostatic potential map determined from an experimental phase image of the Pt/Fe:SrTiO_3_/Nb:SrTiO_3_ sample. The band of pronounced bright contrast arises from the Pt top electrode. The Fe-doped SrTiO_3_ layer and Nb-doped SrTiO_3_ bottom electrode are labelled in the picture. The phase in the substrate region has been flattened to remove the effect of a slight gradient in specimen thickness across the field of view. (The mean inner potential of SrTiO_3_ is calculated to be 15.1 V using neutral atom scattering factors^18^, suggesting that a step in phase of 0.5 radians could be caused by a change in specimen thickness equivalent to 12 unit cells). From the fringe spacing of the electron hologram, the spatial resolution in the phase image is 3.5 nm. The phase noise was calculated by taking the RMS of a profile averaged across 130 nm (360 pixels) and acquired from the substrate region and was found to be 0.03 rads (2π/210). From the error of the phase measurement and specimen thickness, the error on the measurement of the potential can be estimated as ±0.07 V. The region containing the poly-crystalline platinum top contact is difficult to interpret in terms of its mean inner potential due to diffraction effects. However, the specimen has been carefully tilted to minimize the effects of diffraction in the SrTiO_3_ region. Figure 1(b) shows an amplitude image reconstructed from the same hologram. Here, no diffraction contrast is observed in the Fe-doped and Nb-doped SrTiO_3_ layers suggesting that the contrast observed in the phase image is directly interpretable in terms of the contributions from the mean inner potential and from the active dopants. Figure 1(c) shows a line profile of the potential obtained by projecting a region of Figure 1(a), indicated by the white box, over a distance of 130 nm in the direction of the layers. This averaging was necessary to improve the signal to noise. The potential continuously increases over a distance of more than 25 nm before remaining flat across the rest of the specimen. From the top electrode interface across the first 25 nm of the specimen, a step in potential of 0.93 ± 0.07 V is observed. With a thickness of the Fe:SrTiO_3_ layer (top layer, highlighted in gray) of 17.2 nm, it can be seen that part of the change in potential takes place in the Nb:SrTiO_3_ electrode, implying a depletion region extending into the bottom electrode (depletion region, highlighted in yellow). A change in gradient of the measured potential is also visible at the Fe:SrTiO_3_/Nb:SrTiO_3_ interface.

**Figure 2:**
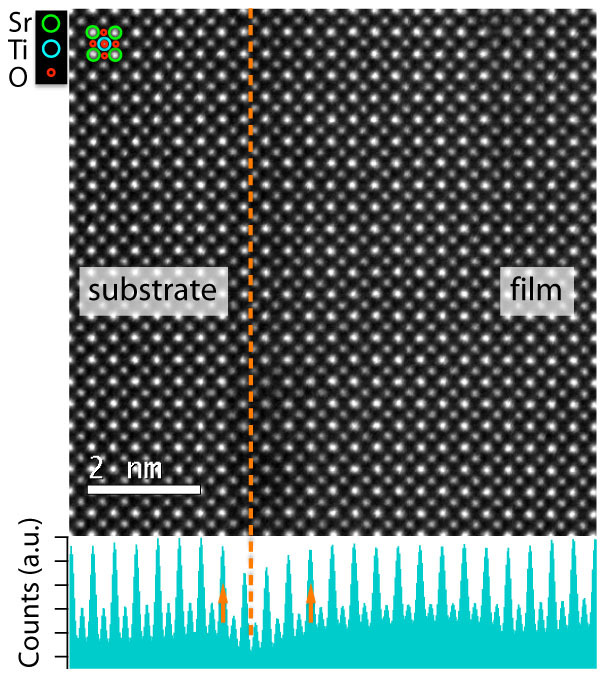
Z-contrast HAADF-STEM image of a Fe-doped SrTiO_3_ (STO) layer deposited on Nb-doped SrTiO_3_. The bottom shows the 1D-averaged profile along the interface, by which the position (dashed line) and sharpness of the interface can be estimated by the negative strain contrast from dechanneling effects.

To obtain information about the structure of the Fe:SrTiO_3_/Nb:SrTiO_3_ interface, high-angle annular dark-field scanning transmission electron microscopy (HAADF-STEM) was performed. Figure 2 shows an atomic-resolution *Z*-contrast HAADF-STEM image recorded from a cross-sectional specimen of the Fe:SrTiO_3_ layer on Nb:SrTiO_3_, viewed along [100] with the interface edge-on. The thickness of the HAADF sample was estimated from an electron energy loss spectroscopy (EELS) spectrum using the log-ratio method and found to be 42 ± 8 nm. A model of a single unit cell of SrTiO_3_ is shown in the upper left corner of the image. The heavier Sr^2+^ ions (*Z* = 38) appear brighter than the lighter Ti^4+^ ions (*Z* = 22). The image confirms that the Fe:SrTiO_3_ film grows epitaxially on the Nb:SrTiO_3_ substrate showing a sharp interface free of dislocations and stacking faults. At the interface between substrates and epitaxially grown films, the displacement of atoms varies along a column, which causes dechanneling effects that reduce or enhance the ADF intensities depending on the inner radius of the detector, giving the so-called strain contrast^22^. The presented HAADF image of the Fe:SrTiO_3_/Nb:SrTiO_3_ interface was recorded under negative strain contrast condition by which the interface can be precisely determined by the minimum of intensity of the 1D averaged profile. The strain contrast appears to extend within 1 unit cell (0.39 nm) into the substrate and within 3 unit cells (1.2 nm) into the film as indicated by arrows in the 1D averaged profile in Figure 2, which might be partially attributed to interdiffusion.

**Figure 3:**
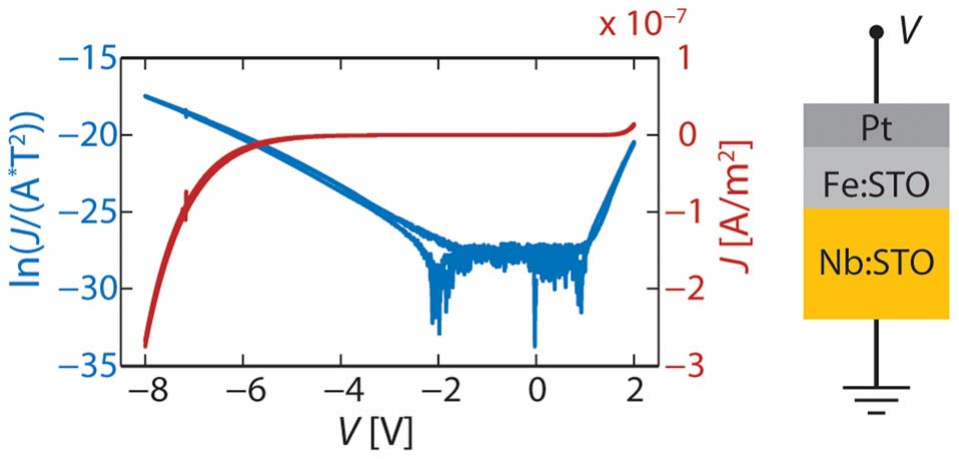
*J-V* curve of the Pt/Fe:SrTiO_3_/Nb:SrTiO_3_ structure.

The current-density–voltage (*J*–*V*) characteristic of the sample is shown in Figure 3 on a linear and a logarithmic scale. The noise visible in the logarithmic curve in the low-voltage range is due to the resolution limit of the measurement setup. The device is highly insulating for low voltages (resistance *R* > 10^8^ Ω) and displays rectifying behaviour. According to the work function differences of the layers, the Pt/Fe:SrTiO_3_ top interface forms a Schottky junction, while the Fe:SrTiO_3_ layer makes an ohmic contact to the Nb:SrTiO_3_ bottom electrode interface. The overall current–voltage characteristics of such a structure would be dominated by the rectifying behaviour of the Schottky contact. The *J-V* relation of our sample with the forward direction being that of the Pt electrode being positively biased with respect to the Nb:SrTiO_3_ electrode confirms that a high Schottky-like barrier exists at the top electrode interface. From the positive branch of the curve, a barrier height of 0.9 eV can be extracted by fitting the curve to the Shockley equation. This value is less than the nominal value of a Pt/Fe:SrTiO_3_ barrier (1.55 eV), but such deviations are typical due to Fermi level pinning caused by defects or metal-induced gap states. The potential profile determined by the electron holography experiment also clearly shows that such a barrier is present at the top electrode interface.

The measured potential step of 0.93 ± 0.07 V is consistent with the barrier height of 0.9 eV obtained from the *J*-*V* curves. However, the overall potential behaviour observed in our samples differs from the one of ideal metal-semiconductor Schottky contacts: As the space charge zone extends over the whole active layer, the barrier is not parabolic in shape. Instead, it is obviously affected by the bottom electrode interface, manifesting itself as a point of inflection in the potential profile at the Fe:SrTiO_3_/Nb:SrTiO_3_ interface (marked 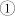 in Figure 1(c)). Associated with this is the fact that the depletion layer penetrates into the bottom electrode, which can be regarded as a second characteristic feature of the measured potential curve (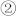 in Figure 1(c)).

## Discussion

To identify possible sources of the observed potential behaviour, a stationary drift-diffusion model is employed to simulate the equilibrium distributions of the electrostatic potential and conduction band profiles resulting from different doping scenarios. SrTiO_3_ can be regarded as a wide band-gap semiconductor and has also been previously studied using drift-diffusion modelling, e.g. to study space-charge effects at grain boundaries^23^,^24^.

## 

We considered a one-dimensional model geometry of length *L* = 100 nm representing a 17 nm thick Fe:SrTiO_3_ layer—corresponding to the I-layer of the MIM structure—and a 83 nm thick part of the Nb:SrTiO_3_ bottom electrode. The types of dopants and their distribution within the structure were taken as parameters in the simulation. Three types of dopants were distinguished: Singly ionizable donors of concentration *N*_Nb_ in the Nb:SrTiO_3_ layer, and doubly ionizable donors of concentration *N*_VO_ as well as singly ionizable acceptors of concentration *N*_A_ in the I-layer. The electrostatic potential *ψ*(*x*) was obtained by solving Poisson's equation 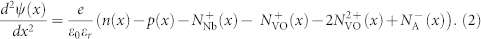
Here, *x* denotes the spatial coordinate, *e* the elementary charge, *ε*_0_ the free space permittivity, *ε*_r_ the relative permittivity, *n* the electron concentration, *p* the hole concentration, and 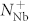, 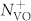, 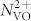, and 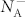 the concentrations of the corresponding ionized dopants. In the following, the notation of the concentrations' and the potential's dependence on *x* will be omitted for brevity. The electron and hole concentrations can be expressed in the form 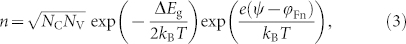

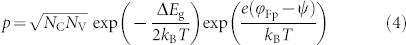
where *k*_B_ is the Boltzmann constant, *T* the absolute temperature, and Δ*E*_g_ the band gap. *N*_C_ and *N*_V_ are the effective densities of states in the conduction band and in the valence band, respectively, and *φ*_Fn_ and *φ*_Fp_ the quasi-Fermi potentials of the electrons and holes, which are constant and coincide in equilibrium state. The concentrations of the ionized dopants are related to the total dopant concentration and the potential via 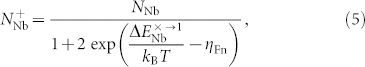

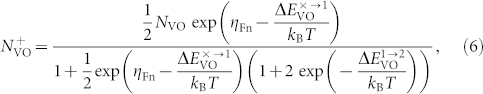

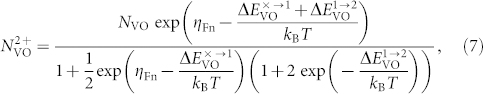

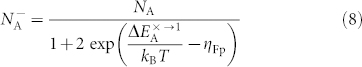
with 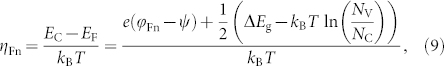

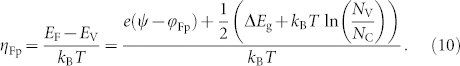
In the above equations, 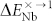, 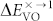, 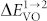, and 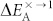 denote the dopant ionization energies, *E*_F_ the Fermi level, *E*_C_ = *E*_C_(*x*) the conduction-band edge and *E*_V_ = *E*_V_(*x*) the valence-band edge. Numerical values of the physical parameters used in the calculations were taken and adapted from Moos & Haerdtl^25^ and are listed in Table 1.

The boundary conditions for *ψ* can be expressed in terms of the barrier heights at the contacts *φ*_B_(0) and *φ*_B_(*L*). If the conduction-band edge at *x* = *L* = 100 nm is defined as the reference point *E*_C_(*L*) = 0 eV, then the potential at this point is given as 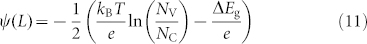
and the potential at *x* = 0 can be calculated as 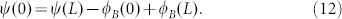
The barrier height *φ*_B_(0) was chosen to be 1 V to represent the Schottky-like contact at the Pt interface, while *φ*_B_(*L*) was chosen to be 0.05 V to model an ohmic interconnect to the bottom electrode. Replacing the charge carrier density terms in (2) with their respective expressions of the form of (5) to (8) results in a non-linear differential equation for the electrostatic potential *ψ*. Along with the boundary conditions (11) and (12), this equation was discretized on the computational domain using a finite-difference scheme and the resulting system of equations was solved in a Newton-Raphson loop. For the given layer structure, conduction-band profiles can be easily calculated from the potential distributions using the linear relationship 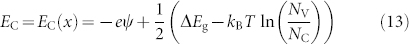
As in all scenarios presented in the subsequent paragraphs the donor concentrations do not exceed the effective density of states in the conduction band, Boltzmann statistics instead of Fermi-Dirac statistics have been used in (3), (4), (9), and (10) for the ease of calculation.

In all our calculations, a homogeneously distributed donor density *N*_Nb_ = 10^20^ cm^−3^ was assumed in the bottom electrode to account for the metallic conductivity of the substrate. Since there is experimental evidence that the nominally acceptor-doped Fe:SrTiO_3_ layer contains a large amount of donor-type oxygen vacancies generated during sample fabrication^26^, different distributions of acceptor-type and donor-type dopants of concentrations *N*_A_ and *N*_VO_ were considered in the I-layer.

**Figure 4:**
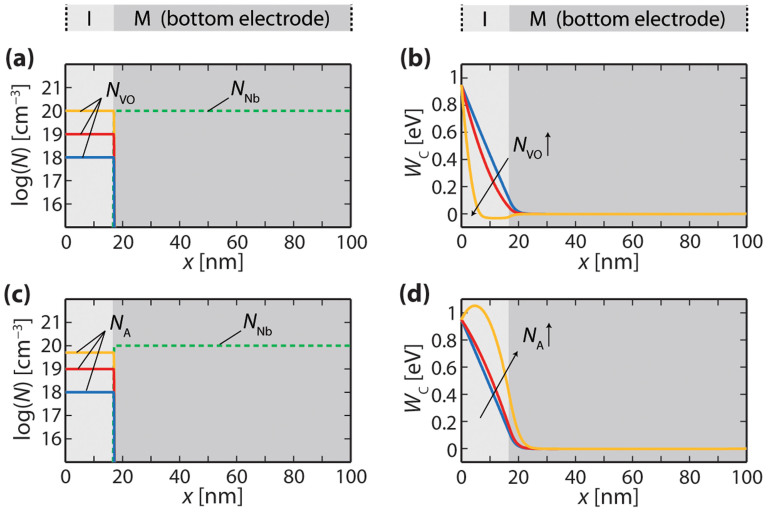
(a) Dopant distributions and (b) resulting energy-band profiles assuming a homogeneous donor distribution in the I-layer; (c) Dopant distributions and (d) resulting energy-band profiles assuming a homogeneous acceptor distribution in the I-layer.

First, the effect of including homogeneous distributions of either donors or acceptors in the I-layer is examined. Figures 4(a) and (b) show the specified dopant distributions and corresponding conduction-band profiles for three different donor concentrations *N*_VO_ = 10^18^ cm^−3^, 10^19^ cm^−3^, and 10^20^ cm^−3^. The conduction-band diagrams indicate that a barrier with a maximum of approximately 0.95 eV forms at the top electrode interface and decays into the I-layer, whereas the bands are flat in the bottom electrode. The width of the barrier decreases with increasing donor concentration *N*_VO_. Its shape is parabolic for high *N*_VO_ and becomes linear for low *N*_VO_ as the high donor density in the bottom electrode *N*_Nb_ restricts the extension of the space charge zone to the I-layer. Thus, no depletion layer develops in the bottom electrode and none of the characteristic features of the measured curve evolves for a purely donor-doped I-layer. Figures 4(c) and (d) illustrate dopant distributions and conduction-band diagrams for three different acceptor concentrations *N*_A_ = 10^18^ cm^−3^, 10^19^ cm^−3^, and 5 × 10^19^ cm^−3^ in the I-layer. For the two lower concentrations (10^18^ cm^−3^ and 10^19^ cm^−3^), a barrier with a maximum at the interface of approximately 0.95 eV develops. For a concentration of 10^18^ cm^−3^, the conduction band decreases almost linearly in the I-layer, whereas for *N*_A_ = 10^19^ cm^−3^ it exhibits a slightly convex curvature. In both cases, the depletion layer does not extend considerably into the bottom electrode. For the highest concentration (5 × 10^19^ cm^−3^), the convex curvature is much more pronounced, leading to a barrier maximum of the conduction band of more than 1 eV at a distance *x* ≈ 8 nm from the Pt electrode interface. In that case, a remarkable part of the space charge zone extends into the bottom electrode. However, the energy-band profile does not exhibit the regions of distinct gradients with an inflection point at the bottom electrode interface that are measured experimentally.

**Figure 5:**
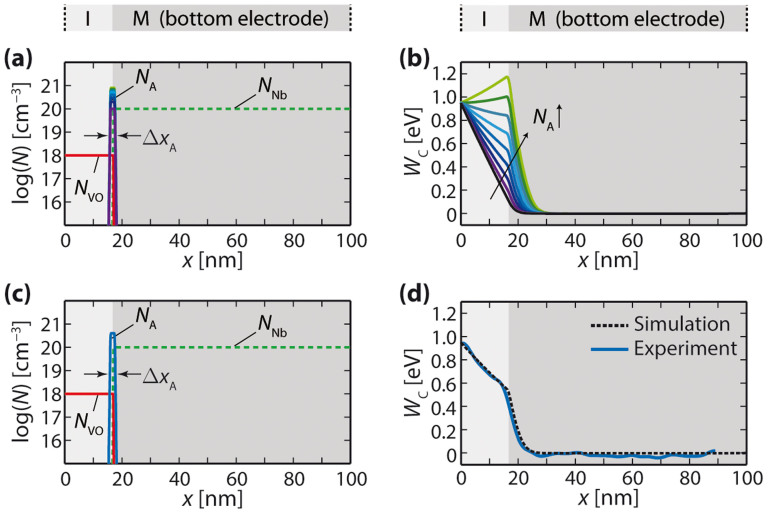
(a) Dopant distributions and (b) resulting energy-band profiles assuming a homogeneous donor concentration in the I-layer and an acceptor-type interface concentration at the Nb:SrTiO_3_ electrode interface; (c) Dopant distributions used to fit the experimental data; (d) Comparison between the simulated and the experimentally measured energy-band profile.

In a next step, we investigate the influence of interface states on the energy-band profile by considering thin layers of acceptor-type dopants at the bottom electrode interface with a donor-doped I-layer (donor concentration *N*_VO_ = 10^18^ cm^−3^). Figure 5(a) shows the dopant distributions for acceptor concentrations varied from *N*_A_ = 1 × 10^20^ cm^−3^ to 8 × 10^20^ cm^−3^ in steps of 1 × 10^20^ cm^−3^. The width of the acceptor distributions is set to be Δ*x*_A_ = 1.5 nm. The corresponding energy-band diagrams are presented in Figure 5(b). The black curve forming an almost triangular barrier corresponds to an acceptor concentration of zero. With increasing acceptor concentration, the conduction band at the bottom electrode interface is shifted upwards, resulting in an inflection point in the conduction-band curve at this position. For high enough concentrations of *N*_A_, the deflection is large enough to yield a maximum of the conduction band at the bottom electrode interface. The upward shift of the energy band involves an extension of the depletion layer into the bottom electrode as well as the formation of distinct potential gradients in the I-layer and the bottom electrode.

Apparently, by introducing acceptor-type states at the bottom electrode interface, the calculated conduction-band profile exhibits the same characteristic features as the measured one: Different gradients on either side of the bottom electrode interface, equivalent to a point of inflection at the bottom electrode interface (feature 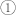), as well as a depletion layer extending into the bottom electrode (feature 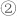). From Figures 5(c) and (d), it can be seen that a good fit to the experimental data is obtained by choosing *N*_A_ = 4 × 10^20^ cm^−3^ and Δ*x*_A_ = 1.5 nm with *N*_VO_ = 10^18^ cm^−3^ and *N*_Nb_ = 10^20^ cm^−3^. Both the gradients in the conduction-band profile and the width of the space-charge zone in the Nb:SrTiO_3_ substrate are well reproduced.

Our findings suggest that acceptor-type dopants are present near the electrode interface between the differently doped SrTiO_3_ layers in the pristine Pt/Fe:SrTiO_3_/Nb:SrTiO_3_ structures. From the point of view of defect chemistry, Sr vacancies are the most probable acceptor-like defects in Nb:SrTiO_3_. Indeed, highly resistive layers have been detected at the surface of Nb:SrTiO_3_ crystals^27^,^28^, which supports the idea of accumulated Sr vacancies at the Nb:SrTiO_3_ side of the bottom electrode interface. Further, it can be seen from Figures 5(a) and (b) that the acceptor concentration at the interface needs to exceed the donor concentration of *N*_Nb_ = 10^20^ cm^−3^ in the Nb:SrTiO_3_ to result in an inflection point. A concentration as large as *N*_A_ = 4 × 10^20^ cm^−3^ provides a good fit to the experimental data (cf. Figures 5(c) and (d)), which is why the observed potential behaviour cannot be solely explained by interdiffusion of Fe into the Nb:SrTiO_3_.

In summary, we analysed the electrostatic potential profile in pristine Pt/Fe:SrTiO_3_/Nb:SrTiO_3_ structures using off-axis electron holography and numerical calculations. The measured potential profile does not show typical Schottky-like behaviour, but exhibits a change in gradient at the bottom electrode interface. The barrier spans the entire I-layer and decays within the first few nanometers of the bottom electrode. The simulations reveal that the presence of acceptor-type dopant concentrations at the Nb:SrTiO_3_ electrode interface in a donor-doped Fe:SrTiO_3_ layer reproduces the characteristic features of the measured potential. A plausible explanation for the interface acceptor concentration are Sr vacancies on the Nb:SrTiO_3_ substrate surface. Based on our findings, we conclude that voltage drops at the bottom interfaces of Pt/Fe:SrTiO_3_/Nb:SrTiO_3_ devices cannot be neglected during electroforming and resistive switching. Thus, the movement of oxygen vacancies in the vicinity of the bottom electrode during resistive switching has also to be considered. Our results suggest that local changes in doping concentration or defect distribution at the interfaces of resistive switching devices can have a significant impact on the local field distribution and the resulting site of oxygen vacancy movement.

## Methods

### Sample fabrication

Epitaxial 1 at%-Fe-doped SrTiO_3_ layers were grown by pulsed laser deposition using a KrF-excimer laser (wavelength 248 nm; frequency 5 Hz; pulse duration 25 ns). Ceramic targets with nominally the same composition have been employed. We used commercially available (001)-oriented 1 at%-Nb-doped SrTiO_3_ substrates (Crystec, Berlin, Germany) that have been annealed for 4 h at 1000°C in air prior to the deposition to ensure a defined, flat step-terrace structure. Thin films have been grown at 700°C in an oxygen pressure of 0.25 mbar with a laser fluence of 0.8 J/cm^2^. After cooling to room temperature in 500 mbar of pure O_2_, the surface morphology and epitaxial character of the films were assessed using atomic force microscopy and X-ray diffraction, respectively^29^. Pt electrodes were fabricated directly on the layer stack using UV-lithography and ion beam etching of a dense Pt film deposited by magnetron sputtering. The thickness of the Fe:SrTiO_3_ layer could be precisely determined to be 17.2 nm from atomic-resolution HAADF-STEM images.

### HAADF-STEM

HAADF-STEM was carried out at 300 kV on an FEI Titan Ultimate TEM equipped with a high-brightness field emission gun and a spherical aberration corrector on the condenser lens system, using a probe semi-convergence angle of approximately 30 mrad. STEM images were acquired from a cross-sectional lamella prepared using focused ion beam (FIB) milling with Ga ion beam at 30 kV beam energy on an FEI Helios NanoLab 400S workstation, followed by thinning with Ar ion milling in a Bal-Tec Res-120 ion beam milling system (beam energy of 2.5 keV, beam current of 1.5 mA, specimen tilt angle of ±12°) and cleaning in a Fischione 1040 Nanomill with Ar ion at beam energy of 500 eV, filament current of 0.21 mA, and specimen tilt angle of ±10°.

### Electron holography

Off-axis electron holography was performed using a FEI Titan TEM operated at 200 kV. In the present study, electron holograms were acquired from parallel-sided specimens of nominal thickness of 200 nm, which were prepared using FIB milling at 8 kV in an FEI Strata 400 Dual-Beam system. Ga implantation into the specimen surface was minimized by drawing a 1-μm-thick line of marker pen ink above the region of interest, which was then covered by a 2-μm-thick layer of W. After FIB milling, the ink was removed using plasma cleaning to leave a region of vacuum for the holographic reference wave. A Lorentz lens was used to achieve an optimal field of view for electron holography with the conventional objective lens switched off. Each hologram was acquired for 16 s to maximize the signal to noise in the phase images, which were converted into maps of the change in potential across the layers using (1). The specimen thickness *t* was measured to be 200 ± 10 nm using convergent beam electron diffraction and assumed to be fully electrically active. As the effects of TEM specimen preparation on the measurement of potentials in SrTiO_3_ have not yet been investigated in detail, it was necessary to perform a systematic study to test the effects of electron beam intensity and specimen preparation on the measured phases. Several different specimens were prepared and observed using different electron beam currents and also using carbon coating to reduce the build-up of charge in the specimens. In addition, the phase measured in the vacuum region was flat which suggests that the specimens were not charging. It is known that for Si, high dopant concentrations of the order 10^20^ cm^−3^ can make artefacts such as the inactive layer and charging negligible^20^. Holograms were acquired from different regions of several different TEM specimens to ensure consistency of the results. For electron holography it is necessary to work away from a crystallographic axis, therefore as the active layers in the specimens are relatively small, it was necessary to ensure that the interface was exactly edge-on in the microscope and that it was not blurred in projection during the acquisition of the holograms.

## Author Contributions

C.L. fabricated the samples and conducted the electrical measurements. H.D. conducted the HAADF-STEM measurements. D.C. conducted the electron holography measurements. A.M. performed the simulations. A.M., D.C., H.D. and C.L. contributed to writing the manuscript. S.M., R.D., R.D.B. and R.W. supervised the research. All authors discussed the results and commented on the manuscript.

## Figures and Tables

**Table 1:** Numerical values of the physical parameters at 300 K

Symbol	Value	Unit	Symbol	Value	Unit
*T*	300	K	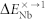	50	meV
*ε*_r_	100		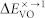	3	meV
Δ*E*_g_	3.1	eV	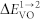	30	meV
*N*_C_	2.1 × 10^20^	cm^−3^	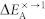	890	meV
*N*_V_	1.3 × 10^20^	cm^−3^			
